# Adolescent and Young Adult Cancer Survivorship Educational Programming: A Qualitative Evaluation

**DOI:** 10.2196/cancer.5821

**Published:** 2017-02-10

**Authors:** Deborah Vollmer Dahlke, Kayla Fair, Yan Alicia Hong, Debra Kellstedt, Marcia G Ory

**Affiliations:** ^1^ Texas A&M School of Public Health Health Promotion and Community Health Sciences Texas A&M University Austin, TX United States; ^2^ TX A&M School of Public Health Health Promotion and Community Health Sciences Texas A&M University College Station, TX United States

**Keywords:** cancer survivorship, adolescent and young adult, qualitative, mixed methods, evaluation

## Abstract

**Background:**

This program evaluation considers the need for increased professional and patient education for adolescent and young adult (AYA) cancer survivorship. Due to the high incidence of late effects of cancer treatment among AYA cancer survivors, knowledge sharing and communications are needed throughout the transition from cancer care into community care. AYA survivors are likely to need developmentally appropriate psychosocial care as well as extensive follow-on surveillance by physicians who are educated and aware of the likely chronic conditions and late effects that may occur in these patients.

**Objective:**

The objective of this study was to evaluate the outcomes of the After Cancer Care Ends, Survivorship Starts for Adolescent and Young Adults (ACCESS AYA) programming. The intent of the ACCESS AYA program was to build health literacy around AYA survivorship issues and to stimulate improved communications between survivors and health care providers. This paper addresses the central research question of “How did the ACCESS AYA program increase health literacy, communications, and understanding among AYA survivors and providers?”

**Methods:**

The primarily qualitative evaluation included a brief introductory survey of participant awareness and effectiveness of the ACCESS AYA project serving as a recruitment tool. Survey respondents were invited to participate in in-depth interviews based on interview guides tailored to the different stakeholder groups. The evaluation used the Atlas Ti qualitative database and software for coding and key word analyses. Interrater reliability analyses were assessed using Cohen kappa analysis with Stata 12.1 (StataCorp LLC) software.

**Results:**

The key themes, which included survivor wellbeing, health care professional education, cancer advocates role and education, hospital and community-based resources, and the role of societal support, are presented in a concept map. The interrater reliability scores (ranging from 1 to minus 1) were .893 for first cycle coding and .784 for the second cycle. In the brief quantitative survey based on a scale of 1 to 5 with 5 as high, the 22 respondents rated their level of awareness of the project with a mean 3.2 (CI 3.02-3.45) and project effectiveness with a mean of 4 (CI 3.72-4.27).

**Conclusions:**

This study contributes to understanding of the ACCESS AYA survivor community in central Texas and the health care professionals and advocates who aid them in their efforts to a new normal life and wellbeing in their survivorship. The results of the evaluation highlight the need to continue to build both survivor and professional resources to address the unique impact of cancer on AYA cancer survivors.

## Introduction

### Overview

In the United States, improvements in overall cancer survival rates experienced by adolescent and young adult (AYA) cancer survivors ages 15 to 39 years have not kept pace with survival rates for adults and pediatric patients. Despite improvements in treatment modalities for many of the cancers that affect AYAs, survival rates for many common cancers experienced by AYAs continue to be concerning [1-6]. Further, AYA cancer survivors face long-term risks from their cancer care, including excess risks of mortality, incidence of secondary primary neoplasms, cardiovascular disease, neuroendocrine and neurocognitive dysfunction, and psychosocial effects [4-7]. Intellectual and psychosocial concerns such as depression and anxiety also affect this group, as they frequently suffer developmental, cultural, and educational setbacks as a result of their cancer treatment [7]. Researchers speculate that the lack of improvement in AYA survival may be due to a combination of factors including deficiencies in care [1,2].

The LIVESTRONG Young Adult Alliance’s position statement on quality care for AYAs suggests that there are gaps in both provider and survivor education to address the unique needs of AYA cancer survivors [8]. Since 2006, with the publication of the National Cancer Institute (NCI) and LIVESTRONG Foundation’s first joint progress review group statement in AYA oncology, AYAs have received increased attention as a population that experiences disparities in care, including poorer survival rates overall than both older and younger cancer patients [1-9]. These reports indicate the need to enhance both quality of life (QOL) and care quality for AYA cancer survivors and call for providers and hospital systems to address the specific health and psychosocial needs of AYAs. To achieve this will require increased education for both providers and survivors. Educational emphases include topics such as the management of cancer survivorship and late effects including awareness of concerns for fertility and body image issues; recognition of the unique context of psychosocial growth and development among AYA survivors; assessment of and attention to cognitive, psychiatric, and psychosocial effects and needs; improved transition to off treatment care, including education of community provider; and referral to available age-appropriate information and support services when indicated [7-9].

Yet, today, few resources exist to train community medical professionals on the unique survivorship needs of AYA cancer survivors. AYA survivorship clinics and educational programs needed to ensure that AYA survivors and caregivers are aware of late effects of treatment and have access to resources to support improved QOL are lacking in most communities [7-9]. This lack of information underscores the need for integrated programs that (1) train providers and educate survivors, (2) establish networks and shared models of care with transition paths from treatment to community care, and (3) build health promotion tools to support improved quality of life among AYA cancer survivors.

### After Cancer Care Ends, Survivorship Starts for Adolescents and Young Adults Program

To meet the need for focused education, the After Cancer Care Ends, Survivorship Starts for Adolescent and Young Adults (ACCESS AYA) program was designed as a strategic combination of provider and survivor education directed at community health care providers, AYA survivors, their families, and cancer patient advocates. The program’s professional medical education was targeted at community and hospital-based family practice and internal medicine physicians and nurses. These are the professionals most likely to provide follow-up medical care to AYA cancer survivors who have transitioned from oncology care into community care [8]. ACCESS AYA focused on 3 elements of professional education: (1) formal, accredited continuing medical education program (CME), (2) a half-day live educational CME session that included case studies and presentations on AYA late effects, and (3) a series of medical briefs, AYA Prompt Evidence Assessment and Review of the Literature Service, known as AYA PEARLS. Examples of the PEARLS are provided in Multimedia Appendix 1.

Feelings of isolation and lack of peer support have been identified as important issues and concerns among AYA cancer survivors, both during their time in treatment and posttreatment [7]. To address this need, the ACCESS AYA program produced 2 annual, half-day interactive, educational sessions for survivors, friends and family, and community cancer advocates. During the project operating period, an estimated 4000 central Texas AYA survivors, 15,000 physicians, and 18,500 nurses across Texas received information about the ACCESS AYA program via mail, email, or print materials. As reported in the project’s final report to the Cancer Prevention and Research Institute of Texas (CPRIT), the project’s funder, direct interpersonal contact was made with approximately 325 AYA cancer survivors; 785 health care professionals, including nurses, physicians, and residents; and over 175 cancer advocates and care givers (ACCESS AYA Final Report, submitted to CPRIT as personal communication by Deborah Vollmer Dahlke, November 2013).

Use of mHealth social and digital media was an innovative element in the survivor public education efforts. The ACCESS AYA grant supported marketing and dissemination of the AYA Healthy Survivorship iPhone app. Over 850 users downloaded the Healthy Survivorship app from the Apple App Store during the project period. The app provides an interactive AYA survivor health and well-being assessment and links to the Children’s Oncology Group’s *Health Links,* several of which are offered both in English and Spanish. Both the iPhone app and its companion website (www.healthysurvivorship.org) offer AYA survivors links to the LIVESTRONG and Journey Forward cancer survivorship care plans. This program was conducted in collaboration with the Communities of Texas Cancer Activity Resource Education Support (CTxCARES), a Centers for Disease Control and Prevention (CDC) Cancer Prevention and Control Research Network–funded project at Texas A&M School of Public Health.

The primary aim of this study was to share results from the evaluation of ACCESS AYA, which addressed several key research questions based on semistructured interviews with 4 sets of stakeholders: AYA survivors, health care providers including both nurses and physicians, hospital administrators, and leaders of cancer survivor advocacy groups. The central research question was “How did the ACCESS AYA program increase health literacy, communications, and understanding among AYA survivors and providers?” The concept of health literacy as used in ACCESS AYA is reflective of AYA survivors’ ability to function in the health care environment and, as such, depends on the characteristics of the AYA survivors, their health care providers, and the health care system in which they operate. Thus, health literacy is a dynamic state that may depend on the awareness of the patients of their medical problems and the concomitant knowledge and awareness of the health care provider. An individual's health literacy may vary depending on care needs, the health care provider’s knowledge, and the policies and procedures and capabilities of the health care system. In the case of AYAs, a major health literacy concern among survivors, providers, and the system is the lack of awareness of late effects of cancer care including medical, emotional, and psychosocial late effects.

Specific subquestions to this inquiry focused on the common barriers that AYA survivors experience and the stakeholders’ perceptions of opportunities for sustaining and expanding AYA survivorship education programs. The qualitative themes and analyses of this study reflect and build upon the findings from the periodic and final quantitative evaluations and reports that were submitted to CPRIT and the Seton Healthcare Family executives. The quantitative assessments were important, as they reported on numbers of survivors and health care professionals served and the types and numbers of print and digital health materials delivered throughout the project period (ACCESS AYA Final Report, submitted to CPRIT as personal communication by Deborah Vollmer Dahlke, November, 2013).

This evaluation seeks to provide deeper insights into what the ACCESS AYA participants valued and to build a richer understanding of what elements of the educational programs were most important across the spectrum of stakeholders. Additionally, the stakeholders’ responses to question about what barriers continue to affect them can help identify areas for additional communication and educational programming. Finally, the themes and areas of discussion for sustainability and future development can be used to inform future system and policy changes.

### After Cancer Care Ends, Survivorship Starts for Adolescents and Young Adults Theoretical Framework

**Figure 1:**
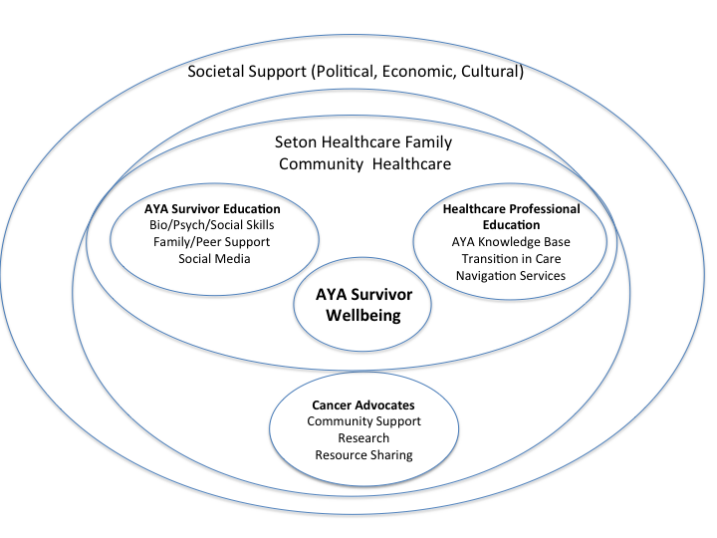
After Cancer Care Ends Survivorship Starts for Adolescents and Young Adults (ACCESS AYA): Theoretical Framework.

The ACCESS AYA program’s educational efforts were focused on improving the AYA survivors’ well-being and supporting changes in their behavior as well as changes in health care professionals’ knowledge and clinical practice behaviors. We anticipated that the effects of the educational programming would extend into the broader clinical, social, cultural, and political environments of the survivors and providers. Based on the social ecological framework for behavioral health by McLeroy et al, the research team identified 5 levels of societal influence in order to construct a theoretical model (see Figure 1) for use in our analyses of the interview narratives [10].

In line with this framework, our social ecological model places the AYA survivors at the center, where physical characteristics, attitudes about survivorship, knowledge, and values exist in relationship to individual health and well-being. The AYA survivors’ educational node in the framework encompasses critical interpersonal relationships with clinicians, parents, partners, friends, and peers, including social media relationships that may influence the survivors’ care and health behaviors both at home and in clinical settings.

On the right side node are the influences of the health care professionals’ education, based on the rationale established in the LIVESTRONG and other AYA studies [7-9] that the knowledge base of physical and psychosocial late effects can influence care and treatment, awareness of transitional needs, and use of survivorship navigation services to support and sustain survivor well-being. The surrounding layer of the Seton Healthcare Family organization and community-based health care represents the organizational norms, culture, and resources of the community health care environment.

In the closer of 2 outer rings, the cancer advocacy groups represent a powerful and contributing sphere of influence in AYA cancer survivorship including physical, financial and social support, research efforts, resource sharing, and dissemination. The final outer ring indicates the levels of societal support for cancer survivor well-being including policies for insurance, financial and social support, and cultural attitudes and values that affect how AYA survivors are perceived and supported or left isolated in the workplace, at school, and in the community.

Each of these levels, or spheres, in the theoretical framework is laden with value judgments of the research team, the interviewers, and the individuals being interviewed. As such, this narrative evaluation of the ACCESS AYA program is naturally influenced by the social context and values embedded in each group as they relay their perceptions of the program effects, barriers, and potential for sustainability.

## Methods

### Subject and Setting

The ACCESS AYA project participants were clinicians, AYA cancer survivors, caregivers, and cancer advocates affiliated with the Seton Healthcare Family in Austin, Texas. The evaluation participants were participants in ACCESS AYA project activities who were invited to compete a brief study survey and to participate in in-depth interviews.

### Research Approach

The criteria and approach for this qualitative evaluation are based in the constructivist models suggested by Guba and Lincoln with criteria including [11,12]:

- Credibility (ie, faithful descriptions or interpretations of human experiences)
- Fittingness (ie, how a study findings fit outside the study and if viewers will find the evaluation results meaningful in their own experience)
- Auditability (ie, if the study is detailed in such as way that it can be replicated)

Audibility can be enhanced through description of the project and clear explanations and justification of (1) study rational; (2) articulation of the researchers’ views on the subject; (3) purposes and goals of the study; (4) description of participant engagement; (5) mutual influences among the researchers, participant, and stakeholders; and (6) explicit details of data collection, analyses, and transformation [12]. Using these criteria as guidelines and as a statement of the evaluators’ philosophical approach to the evaluation, the remainder of this report describes the data analyses, findings, and results and a discussion of future directions.

### Sampling Methodology and Survey for After Cancer Care Ends, Survivorship Starts for Adolescents and Young Adults Evaluation Participation

**Figure 2:**
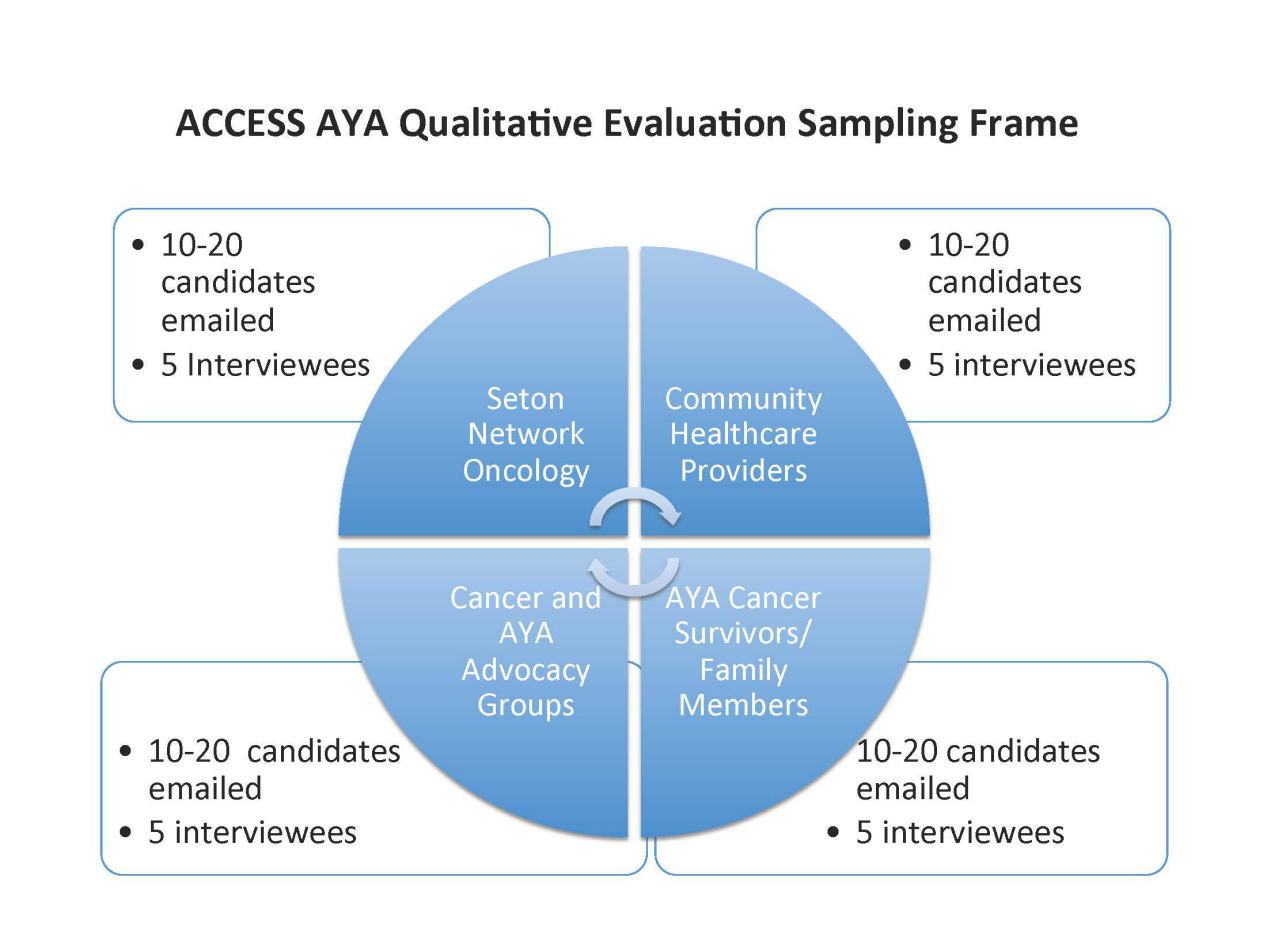
After Cancer Care Ends, Survivorship Starts for Adolescent and Young Adults (ACCESS AYA) sampling frame.

The sampling methodology, described in the sampling frame in Figure 2, was designed to include approximately 20 participants, 5 from each of the following groups: (1) health care administrators and executives from Seton Network Oncology; (2) community health care providers (ie, doctors and nurses); (3) AYA survivors, caregivers, and family members; and (4) community cancer advocates. The rationale for including these groups in the sampling frame is that they reflect the groups of participants in the ACCESS AYA program. These groups are also described in the project’s theoretical model (Figure 1).

The initial contact with the participants was via an email that included a survey to ascertain their willingness to participate, an online consent process, and information on how to contact informants who agreed to participate in the evaluation process. This survey also assessed respondents’ awareness of the ACCESS AYA programs and their perceptions about program effectiveness using a 5-point Likert scale. In addition to the email request, a request for interested AYA survivors to participate in the research study with a link to the survey was posted on a Facebook page operated and maintained by central Texas AYA survivors.

### Evaluation Team and Interview Guides and Methodology

The evaluation team included 4 investigators: 2 investigators conducting the interviews and 2 different investigators analyzing the raw data and providing the coding and analyses of the materials. The coding was done under the guidance of an experienced qualitative researcher, and all members of the team have experience in health behavior research in cancer survivorship. A general interview guide was developed and tailored with questions specifically relevant to each type of participant. The semistructured interview questions were designed with reference to Stufflebeam’s context input process product (CIPP) model of evaluation practice [13,14]. The CIPP model considers evaluation an essential component of improvement efforts and adapts well to qualitative evaluation of programs like ACCESS AYA where there is a need to include context, input, process, and impact statements with deep engagement of a variety of stakeholders.

The interview guide questions for health care professionals and AYA cancer survivors are provided in Tables 1 and 2, respectively. The researchers and research assistants individually or jointly conducted 20- to 30-minute telephone interviews with study participants. Joint interviews were held to assist the interviewers in taking notes as well as in making a recording of the interview. In these situations, 1 interviewer led with questions while the second interviewer listened in via speaker phone. All telephone interviews were recorded with the participant’s agreement, and each participant confirmed they had understood and agreed to the consent process. Once consent agreement was confirmed, the interviewers no longer used the participant’s names so that the recorded and transcribed interviews would remain anonymous to the research team coding the interviews.

The researchers conducting the interviews were experienced health behavior professionals who sought to apply an open and receptive aspect to accommodate positive, neutral, and negative attitudes articulated by the participants. The tape-recorded interviews were transcribed by an external contractor and returned as text documents. The transcribed interviews were coded by the type of participant (ie, physicians, nurse, hospital executive, advocate, or AYA survivor). In cases where a participant had more than one role (eg, both survivor and advocate), the interviewers asked the participant to respond to the questions specific to 1 role, to the greatest extent possible.

**Table 1:** Health care professional interview guide.

Question	Interview questions
1	In what ways have Seton’s professional and patient education programs on AYAs helped you understand the needs of these survivors?
2	In what ways have you shared information on the ACCESS AYA cancer survivorship professional or patient education program with your colleagues or staff?
3	In what ways, if any, has information or education materials about AYA cancer survivors changed the way in which you do your job?
4	What barriers or challenges are you aware of regarding how AYA cancer survivors are cared for or treated in central Texas?
5	Has the information or education regarding AYA cancer survivors changed how you think about or treat other cancer survivors? If so, can you provide examples?
6	What opportunities or challenges do you believe exist in sustaining or expanding programs for educating AYA cancer survivors and their families/caregivers?
7	Do you have any additional thoughts or information you would like to share as part of this evaluation of the ACCESS AYA grant?

**Table 2:** Interview guide for cancer survivor, family member, or caregiver.

Question	Interview questions
1	In what ways have Seton’s education programs on AYA cancer survivorship helped you as a survivor (or as a caregiver or family member)?
2	Have you shared any of the information or educational materials, including the videos or the AYA iPhone app with other AYA survivors, caregivers, or family members? Can you share a story?
3	In what ways, if any, has information or educational materials about AYA cancer survivors helped you (for example, learning about healthy diets or physical activity for AYA survivors)?
4	What barriers or challenges are you aware of regarding how AYA cancer survivors are cared for or treated in central Texas?
5	Has the information or education regarding AYA cancer survivors changed how you think about or treat other cancer survivors? If so, can you provide examples?
6	What opportunities or challenges do you believe exist in sustaining or expanding programs for AYAs?
7	Do you have any additional thoughts or information you would like to share as part of this evaluation of the ACCESS AYA grant?

Once transcribed, the interview narratives were read and checked for accuracy by the first author and a research assistant prior to coding. The electronic files were loaded into Atlas.ti version 7 (Atlas.ti GmbH) for coding and analysis.

The descriptive coding and framework followed fundamental approaches of identifying themes, developing codebooks, and constructing models guided by the theoretical frameworks provided by Miles et al and Saldaña and assessed statements about the merit, worth, satisfaction, and significance of the educational programming for the evaluation [15,16]. These themes were present in the interview guide questions, thus supporting efforts to code statements in interviews to specific themes. In the first cycle of coding, both descriptive and in vivo codes were applied. The coding process started with both researchers independently reading the transcripts and then discussing early findings. First cycle coding themes were developed independently from the interview guide, and additional themes emerged during the second cycle coding process.

Memos were inserted into the Atlas.ti database. Data analysis for the evaluation was informed by an analytical approach suggested by Creswell in efforts to grasp the themes and essential meaning of the stakeholder comments [17].

During the first and second stages of analysis, both research team members independently coded and met with a senior researcher to discuss findings. Any differences or disagreements in coding or thematic analysis were resolved through discussions among the research team members. The interrater reliability scores (ranging from 1 to minus 1) were .893 for the first cycle and .784 for second cycle. The Cohen kappa scores were derived using Stata 12.1 statistical analysis software (StataCorp LLC). Quotations from the stakeholders were further categorized based on coding domains associated with the evaluation’s theoretical framework. As a third stage, a concept map was generated using the codes and themes generated by both first and second cycle coding. Concept maps can provide a useful alternative to code and word-based text analysis in response to open-ended survey questions [18].

## Results

### Overview

A total of 22 participants responded to the study evaluation survey. However, only 18 participated in the qualitative interviews (scheduling conflicts accounted for loss of 3 subjects, and 1 participant declined to be interviewed). The available demographic information was limited by the personal data collection requirements established under the evaluation institutional review board (IRB). Among the 5 participants from the Seton Network Oncology practice, a male nurse, a female AYA nurse navigator, a male medical oncologist, a male palliative care physician, and a female internal medicine physician were interviewed. Among the 4 participants from community health care were a female cancer administration hospital executive, a male cancer program manager, a female community-based surgical oncologist, and a female dietician who worked with cancer patients. The cancer advocacy participants, all of whom were also cancer survivors, included 3 female advocates and 1 male advocate from community-based cancer advocacy groups. The AYA cancer survivors included 4 AYAs (2 males and 2 females) and one female AYA caregiver.

Table 3 provides the results of the initial email survey and the methodology used to assess the participants’ perceptions of the program and recruit participants for the telephone surveys.

**Table 3:** Survey of awareness and effectiveness of the After Cancer Care Ends, Survivorship Starts for Adolescents and Young Adults (ACCESS AYA) program (N=22).

Question	Mean^a^	Standard error	95% CI
Level of awareness	3.2	0.098	3.02-3.43
ACCESS AYA program effectiveness	4	0.132	3.72-4.27

^a^The response scale was 1 to 5, with 5 as the high score.

### After Cancer Care Ends, Survivorship Starts for Adolescents and Young Adults Coding Results and Concept Map

Table 4 provides examples of the first cycle descriptive codes and their relationship to the theoretical model. Focal areas for the first cycle code reflect perceived participant areas of concerns and needs from the interview transcripts.

**Table 4:** List of first cycle codes and focal areas.

Framework region and first cycle descriptive codes		Focal area
**AYA survivor well-being**		
	Barriers to care and lack of access to care	Physical concerns
	Awareness of late effects	
	Use of care plans	
**Educational needs**		
	Personal reflection on survivorship	Psychosocial concerns
	Need for community and peer sharing	
**Needs of daily living**		
	Costs of past care	Financial and insurance concerns
**AYA survivor education**		
	Need for survivorship education	Health literacy
	AYA use of apps and digital technology	
	Use of survivorship plans	
**Information-sharing practices**		
	AYA self advocacy	Training and education
	Lack of ability to communicate with physicians	
**Health care professional education**		
	Age-appropriate care	Survivor education and training
	Lack of awareness of late effects	Provider education and training
	Lack of knowledge of AYA needs	Provider time constraints
	Lack of knowledge of Seton AYA clinic	
	AYA population sparseness and fragmentation	
	CME uptake and professional education programs	
**Survivorship clinic**		
	Referrals and transitions in care	Insurance coverage concerns
	Coordination with navigators	
	Use of survivorship plans with patients	
**Cancer advocates**		
	Advocates role in information sharing	Information gathering and sharing
	Attitudes about AYA research	Delivery of resources
	Lack of knowledge of Seton and other community programs	
	Family and caregiver needs	
	Lack of survivorship care plans	
	Lack of information for nonmedical needs	
**Seton Healthcare Family and community physicians**		
	Impact of AYA educational programs	Financial and human resources
	Improved knowledge of Seton AYA program	Sustainability
**Political, economic, and cultural societal support**		
	AYA political advocacy	Resources
	American College of Surgeons requirements for survivorship care plans	Influence and power
		Practice change

**Figure 3:**
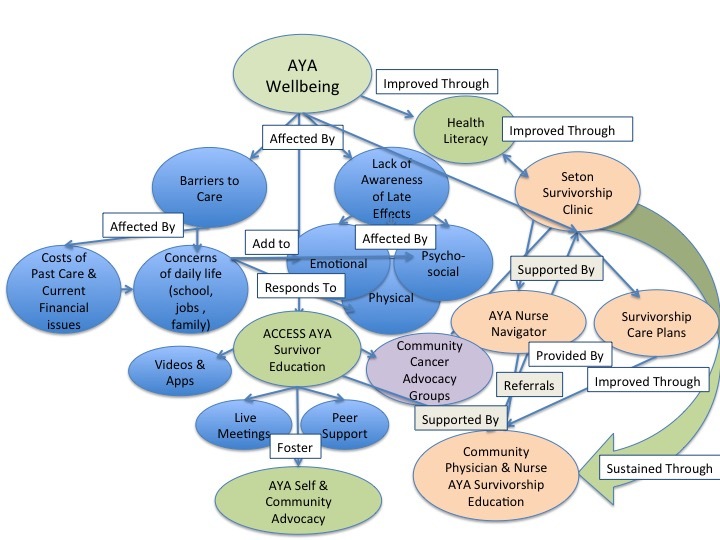
After Cancer Care Ends, Survivorship Starts for Adolescent and Young Adults (ACCESS AYA) evaluation concept map.

The results of the second cycle coding are represented in the ACCESS AYA evaluation concept map (Figure 3). These were generated using the codes and themes from the second cycle coding. In the ACCESS AYA map, the major themes and aims of the program are explored including survivor well-being, the use of survivor and provider education to support health literacy, and communication. An unexpected consequence of the ACCESS AYA programing that emerged as part of the evaluation was the increased desire among AYA survivors to engage in self and community advocacy.

### Illustrative Quotes from the After Cancer Care Ends, Survivorship Starts for Adolescents and Young Adults Evaluation Interviews

#### Well-Being

As mapped out in Figure 3, our evaluation interviews indicated that for many AYA survivors, their concerns about debt from the cost of their care and the economic impact that cancer has had in their lives affect their overall well-being. Some survivors indicated that their financial status was a barrier to their adherence to follow-up care and care for late effects. AYAs also expressed concerns about how their cancer experience affected their ability to function in their daily lives at school, work, and in relationships.

A young brain cancer survivor shared her frustrations about the transition from being in treatment to the new normal of survivorship and her concerns about the ongoing financial costs of cancer care and survivorship.

What would be really helpful is to figure out financial help because that’s kind of one of the big things. It just costs so much for all the treatment . . . the biggest thing is trying to get back to normal routines because you’re used to just being home and dealing with your sickness.

AYA survivor and program participant

For AYA survivors, the concept of well-being is transient, and late effects of care may affect them emotionally as well as physically and mentally [19,20]. They may struggle with the affects of their treatment across all the areas of the social ecological framework, physically, intellectually, socially, and financially. Several of the ACCESS AYA evaluation participants expressed concerns about the effects of their treatment on their mental capacity and worried about how that might affect their future employment and educational opportunities. Well-being among survivors was also expressed in changed awareness and increased empathy for those they encounter.

A lot of people might not even realize how sick people might be and not even look it. I think my experience has made me more aware and less judgmental. [AYA survivor, ACCESS AYA participant]

When asked to address the benefits of the ACCESS AYA program, several survivors commented on the value of being more informed and connected to the community of AYA survivors. According to Sansom-Daly and Wakefield Schroevers, positive social support is strongly protective against the distress and depression that may affect AYA cancer survivors, and many AYAs suffer from post-traumatic stress conditions [20].

I appreciated the connection point, to meet some more people . . . doctors are brilliant and all, there are things that they simply don’t understand because they’ve never been through it . . . there’s a difference between science and experience.

AYA survivor/ACCESS AYA program participant

#### Adolescent and Young Adult Survivor Education

The ACCESS AYA educational programming for survivors covered medical and clinical issues, survivor advocacy, self-efficacy, and opportunities for social engagement with other survivors in real time and in virtual online space. Both patient and professional education programs stressed the importance of the development and use of survivorship care plans as a way to improve health literacy among both the providers and survivors.

My memory is really, really bad, so it *[the care plan]* helps me to have a lot of information to hand over to my doctors. I have probably 8 to 12 different medical people trying to keep me well and going. So, it’s hard to keep up with all that. It helped me along the way when I can’t remember stuff. [AYA cancer survivor, ACCESS AYA participant]

The ACCESS AYA Summits were half-day meetings designed to provide opportunities for interactions with peers, health care professionals, and community cancer advocates. The agendas included a variety of interactive elements including physical activity, cooking demonstrations, and physician presentations on screening and surveillance for second cancers and late effects such as cardiotoxicity.

. . . some of it has been some good practical stuff on how to deal with finances, emotions, the insurance, second opinions, keeping records. My favorite part, honestly, is that it connects you to other people, both experts in the medical field and other people who have been through it . . .

AYA cancer survivor/ACCESS AYA program participant

#### Adolescent and Young Adult Advocacy

A consistent theme throughout the ACCESS AYA education effort was the importance of self-advocacy and advocacy training. The educational seminars included survivor-led discussions on self-advocacy in dealing with the medical community and in life situations as well as engagement in social advocacy for AYA survivorship concerns.

I think that the benefit of a young person understanding and knowing that they are actually part of a larger community, they’re not alone, that they’re part of this community, they’re part of something bigger and they can make a difference, I think is incredibly powerful and can be helpful to their own sort mental and emotional healing. [AYA cancer survivor/cancer advocate]

The shared passion and desire to participate in social advocacy among the AYA survivor community is perhaps an unintended consequence of the ACCESS AYA educational program. Several of the AYA survivor participants stated that as a result of learning about national AYA advocacy organizations in the ACCESS AYA programs like Critical Mass and the OMG Stupid Cancer Annual Conference, they are now participating in advocacy at a national level.

#### Health Care Professional Education

Education of health care professionals appeared to be one of the more challenging aspects of the ACCESS AYA program. The initial plan of offering free online and digital video disc (DVD) CME materials to physicians and nurses was deemed successful only for the nursing professionals. Despite multiple attempts to deliver the CMEs to physicians, uptake was minimal. The innovation of creating the Prompt Evidence Assessment and Review of the Literature Service (PEARLS), both as 1-page briefs and short YouTube videos that included cases and evidence-based facts on AYA survivorship, offered improved dissemination of the professional education materials. Over 450 PEARLS packets containing 3 1-page PEARLS, DVDs with the AYA CME courses, and materials on Seton’s AYA survivorship clinic and navigation were delivered to central Texas physician offices and clinics, and 345 physicians and nurses participated in clinic discussions about the PEARLS’ content.

The PEARLS were delivered both as links from the Seton Survivor Center website and delivered directly to clinics and offices with brief presentations to the clinical staff. A qualitative assessment of the PEARLS dissemination effort is reported on elsewhere. A community physician commented on the difficulty of continuing education and the PEARLS as a delivery mechanism.

So, the education probably has to come case-by-case. That is the way most of us learn anyway. A lot of people are getting a lot of education off emails, webcams and this kind of short vignette.

Community physician

There were differing perceptions in the value and opportunity for providing physician education, as is evidenced by comments from a second community-based physician.

I think it’s a challenge, frankly, to educate any professional once they’ve finished their training. I just think that a lot of people are so busy and so overwhelmed with just workload that taking time for professional education that isn’t mandated by their specialty board, it’s just not going to happen. [Community physician]

A cancer survivor advocate, who also served on the ACCESS AYA advisory group, had a differing opinion regarding health care professional education.

I think educating professionals is a real problem in the young adult community. Because the young adults patient population is fragmented between adult and pediatric and community and academic, I think anything that we can do to break down those walls is what we have to do to move the field forward and to improve the care and treatment of these young adult patients.

Cancer advocate and ACCESS AYA advisory board member

Despite these concerns, there was dispersion of the professional training through the system as evidenced by resident training programs for AYA cancer survivorship provided by a Seton staff physician and via comments from both nurses and physicians about sharing the ACCESS AYA materials with staff and colleagues.

Concerns for the complexity of care of AYA patients and comments about the need for better transitions of patients from cancer care to community care were themes in the health care professional interviews. Both physicians and nurses expressed concerns about lack of time for education as well as the relatively few numbers of AYA survivors among their practice populations.

In addition to delivering information and education, an ACCESS AYA goal was practice change in health care. A community-based palliative care physician reflects on changes in her practice behavior as a result of the ACCESS AYA programming.

I’ve tried to be more deliberate about preparing patients for survivorship while they’re in treatment. I think systematically what we used to do is treat the patients, and then be a little befuddled as to why they weren’t feeling great afterward, either physically or emotionally or both. I've started to be more deliberate about trying to prepare patients for when they finish treatment . . . I have gotten more tuned into the need for behavioral health support for patients who are not yet in survivorship . . . the bigger questions of meaning and comorbid mental health problems are harder, a lot harder. [Community palliative care physician]

According to the views of both the health care professionals and health care administrators, the ACCESS AYA program was successful in creating the content and materials for professional education but struggled in dissemination and adoption. The delivery of the video and print PEARLS were perhaps the most successful elements of the program in that they delivered evidence-based information in a timely and succinct manner and required little investment of time from the health care providers.

#### Community Cancer Advocacy Groups

Cancer advocacy groups and advocacy leaders frequently take on the role of bridging between the medical community and the patients and their families. They are frequently supported both financially and through provider provision of education programs and training in the community by hospital systems and community physicians. Modeled partially on the success of breast cancer advocacy, AYA advocacy groups work to ensure that the unique medical, psychosocial, supportive, and educational needs of teenagers/AYAs living with cancer are met. The roles of advocacy groups include bringing individuals interested in change together and providing coordinated education and support services as well as policy analysis and response. Increasingly, AYA cancer advocacy groups deliver the bulk of their services through social media [21]. Much of the focus of the national AYA advocacy groups is to bring researchers together with survivors to support increased recognition of the unique needs of this population including developing specialist facilities for treatment and survivorship, addressing concerns for delayed diagnosis, and seeking to improve access and quality of care. Central Texas is home to both the national headquarters of the LIVESTRONG Foundation, with its strong focus on AYA survivorship, and the newly formed Critical Mass AYA advocacy group.

I think that it is not unique to central Texas. I think that a challenge that is faced everywhere is this fragmentation of the young adult patient population and the difficulty in breaking down silos of their care and treatment and service. I find that so often the frustration is people don’t get me, they don’t understand what it’s like to be a young person with cancer. Why am I getting materials for old people? It’s different to be in my position. This gives rise to the isolation and the fact that you don’t have anyone, if you’re socially isolated, to process your experience with.

AYA survivor and cancer advocate

A consistent theme among the cancer advocates was their role in the community in sharing and distributing educational resources and programming both via social media and in print and at meetings [21,22]. Several of the cancer advocates participated in the 2 AYA annual summits held during the project and used the venue to both distribute their own information and gather other resources for sharing with their constituencies.

Among the most powerful elements in programs like ACCESS AYA and the Seton Cancer Survivor Center as well as among the advocacy groups are the creation and support for shared communication among of AYA survivors [21,22]. The online Facebook and in-person support community were primarily a creation of the Seton Cancer Survivor Center, but they also reflect the increased emphasis on survivor education and communication from the ACCESS AYA grant efforts. The engagement of the AYA survivors in group meetings further demonstrates the development of a sustainable community engaged in sharing resources, wisdom, and information.

I think a lot of people really identified with that because they were able to hang out with people that had, I guess, maybe the same limitations . . . or similar backgrounds to them and they felt more comfortable . . . They really seemed to enjoy the fact that it wasn’t all based on the illness or the complications . . . it was based on having fun, being normal and moving on . . . [AYA cancer survivor/ACCESS AYA participant]

#### Sustaining After Cancer Care Ends, Survivorship Starts for Adolescents and Young Adults Educational Programs

Programs like ACCESS AYA face challenges in efforts to sustain and expand their reach due to competition for funding and ongoing challenges in hospital and health care operations. When asked about their thoughts regarding sustainability, most respondents mentioned the competition for funding. However, there are valuable insights regarding what it will mean to sustain survivorship education efforts in emerging areas such as caregiver support and palliative care both for pain management and end-of-life care.

To make an analogy . . . we prep people for a hurricane. We take care of people during the hurricane, and we may provide some emergency services after the hurricane, but . . . we don’t help people rebuild when that hurricane is all through . . . I look at caregivers as a patient population that’s emerging and that we are ill-equipped to care for.

Community palliative care physician

## Discussion

### Principal Findings

The results of the evaluation indicate that the program was perceived in a positive light by the members of the representative stakeholder groups interviewed—AYA survivors, clinical health care professionals, administrative health care professionals, and cancer advocates. However, some of the physicians claimed to have not been fully informed of the program and others indicated difficulty in finding time for educational activities given their patient load and clinic demands. Among cancer advocates, there were concerns about the need for additional and ongoing dissemination of the educational materials. Among survivors, most indicated benefits from both the educational program and the navigation and care plan provision services provided by the Seton Survivor Center.

The survivor benefits were in the domains of increased awareness of late effects, use of the app and social media, and increased peer support and engagement. The AYA survivors also indicated increased self-efficacy both for their engagement with physicians and in health care settings and in policy advocacy for the regional and national AYA survivor community.

Among physicians, nurses, and health care administrators, there was clear evidence of increased knowledge of AYA health and psychosocial concerns and greater awareness of the unique needs of the AYA population. There was evidence of practice change in the way nurses and physicians treated and perceived survivor posttreatment needs, both physical and psychosocial. The high level of effectiveness and value of the nurse navigator and staff of the Seton Survivor Center were remarked upon by both survivors and providers. While the nurse navigator was not directly funded by the CPRIT grant, her engagement in the project as an advisor and collaborator was an important element in the success of the education programming.

The ACCESS AYA program appears to have succeeded in increasing awareness of AYA survivors as a unique population and building a sense of community among AYAs, their caregivers, and advocates. The survivors’ self-avowed increased social and political awareness and desires for activism is also an indicator of increased self-efficacy. An unexpected consequence of the ACCESS AYA programing that emerged as part of the evaluation was the increased desire among AYA survivors to engage in self and community advocacy.

These elements tie to the societal support realm in the evaluation’s theoretical framework related to building skills and support for political, economic, and cultural aspects of AYA survivorship. Both the cancer advocate and AYA survivor interviews indicated that the participants found value and benefit in the increased sense of community and the potential to take action based on information and education provided by the ACCESS AYA program. There were also indications among the health care professionals that increased advocacy and self-management both for patients and their families was a positive benefit of the ACCESS AYA programming.

ACCESS AYA was designed to address both knowledge gaps and service delivery gaps among AYA cancer survivors and providers. The knowledge gap includes the lack of information and awareness among AYA survivors and providers about the characteristics that make this population unique among cancer survivors. This includes lack of knowledge about disparities in survival, increased mortality, greater incidence of second cancers, awareness of late effects of treatment, and psychosocial concerns that affect quality of life among AYAs. The lack of service delivery includes the lack of age-specific clinics for both cancer care and posttreatment care and programs. The stakeholder groups in the evaluation shared perceptions that were unique to their experience, some reflecting on the ACCESS AYA materials and others on AYA survivorship concerns in general. The delivery gaps identified by the stakeholders suggest opportunities for increased information and resource sharing among health care professionals, both oncologists and community providers as well as among the survivor and advocate stakeholder communities. This finding is supported by Zebrack in his analysis of the service needs of AYA survivors [8]. Across all of the stakeholders, there was general agreement on the importance of programs and educational efforts to ensure the well-being of the survivors. Similarly, there was consensus for the need to building a knowledge base and a community repository of resources to support AYAs in their survivorship efforts. AYA survivor needs regarding information sharing, especially among peers, were assessed in research by Freyer [23]. Among the survivors and cancer advocates, there was acknowledgment and support for increased social support and peer engagement, which was identified as one of the key research gaps in a recent National Cancer Policy Forum Workshop held jointly by the LIVESTRONG Foundation and the Institute of Medicine [24].

### Limitations

We note that there are limitations in our qualitative approach to evaluating ACCESS AYA. While one of the strengths of qualitative research is the “making of meaning,” the meaning is subject to the authors’ understanding and interpretation. Among the limitations inherent in this study are the small sample size and potential of researcher prejudice and bias, observer effects, and the authors’ ability to present the research in such a way that it could be replicated in the future.

Qualitative analyses and evaluations allow us to share the voices of the stakeholders and participants from an interpretive perspective. In considering the limitations in this evaluation, the research team attempted to recognize the subjectivity of their lenses in viewing the ACCESS AYA project. The selection of the interview participants may be perceived as a limitation, as they were self-selected. The participant sampling frame was well reasoned, and the inclusion of groups of AYA survivors, health care professionals, and advocates was highly relevant to the evaluation research. The views expressed by the AYA survivors may not reflect the perspectives of AYA cancer survivors who prefer to forget about their cancer experience or those who are less affected by late effects of treatment. And, certainly, the specific geographic region of central Texas may limit the generalizability of the research, although the program delivery was diverse and included racial and ethnic groups as well as gay, lesbian, and bisexual AYA survivors.

The assumption was that data collection via a brief phone interview was appropriate for addressing the research objectives, and yet in hindsight this may not have resulted in as rich data responses as longer face-to-face interviews. The limited time for some of the phone interviews was driven by the time constraints of the health care professionals. Limitations may exist in the narrow use of interviews as the primary source of data. However, the research team was familiar with the print and video materials of ACCESS AYA, and team members participated in field observations, providing additional richness and robustness to the evaluation analysis. Finally, the results and data must be appropriately analyzed and the findings adequately corroborated by using multiple sources of information.

### Conclusions

Qualitative studies such as this evaluation have the potential to complement quantitative evaluations by bringing to the forefront the multiple realities of the various stakeholders. The values and benefits of the program evaluated reflect the realities of the lives and work of the participants. What worked in ACCESS AYA and what challenges and opportunities remain are articulated through the voices of those most affected.

In responding to the evaluation’s primary and secondary research questions regarding the value and benefits of both AYA survivor and professional education, we suggest that overall ACCESS AYA was moderately successful in reaching its intended population but that additional work is needed to continue the educational efforts.

The evaluation and the ACCESS AYA program were built on an action agenda for change through education and information in the way AYA survivors perceive themselves and are perceived by their peers, providers, advocates, and communities. The agenda for change includes ongoing developments in the skills and knowledge base of community health care professionals, doctors, nurses, and administrators who treat and care for AYA cancer survivors.

This evaluation offers a contribution to the understanding of the AYA survivor community and to the health care professionals and advocates who aid them in their efforts to a new normal life and well-being in their survivorship. This evaluation highlights the need to continue to build the survivor and professional resources to address the unique impact of cancer on the quality of life and well-being of AYA cancer survivors. To adequately provide quality care for AYA survivors, health care organizations and providers must address both the health and the psychosocial needs of this population. To do so will require ongoing research in understanding AYA survivors as a highly heterogeneous population that requires management of cancer and treatment late effects including fertility, body image, and cognitive and most particularly psychosocial effects and care needs. These areas of research have been identified and expanded upon in the increasing body of knowledge regarding AYA cancer care and survivorship [21,25,26]. As part of this process, policy and programmatic improvements are needed to facilitate transition to AYA survivors into community and off treatment care through the provision of care plans and age-appropriate information and support service resources [26].

The development of survivorship research methods and measurable outcomes to support evidence-based educational materials and guidelines depends on the availability of funding opportunities at a time of increasingly limited resources and economic pressures in both academic and health care settings. The ability to develop quality research studies related to the AYA population is also dependent on the recruitment of sufficient numbers of survivors into these studies.

### Multimedia Appendix

Multimedia Appendix 1. Examples of After Cancer Care Ends, Survivorship Starts for Adolescent and Young Adults (ACCESS AYA) Prompt Evidence Assessment and Review of the Literature Service (PEARLS).

## Appendix

Multimedia Appendix 1 Examples of After Cancer Care Ends, Survivorship Starts for Adolescent and Young Adults (ACCESS AYA) Prompt Evidence Assessment and Review of the Literature Service (PEARLS).

## Abbreviations

ACCESS AYA: After Cancer Care Ends, Survivorship Starts for Adolescent and Young Adults
AYA: adolescent and young adult
CDC: Centers for Disease Control and Prevention
CIPP: context input process product
CME: continuing medical education
CPRIT: Cancer Prevention and Research Institute of Texas
CTxCARES: Communities of Texas Cancer Activity Resource Education Support
DVD: digital video disc
IRB: institutional review board
NCI: National Cancer Institute
PEARLS: Prompt Evidence Assessment and Review of the Literature Service
QOL: quality of life
