# Supplementary material for: Adolescent and Young Adult Cancer Survivorship Educational Programming: A Qualitative Evaluation
Source: JMIR Cancer. 2017 Feb 10;3(1):e3. doi: 10.2196/cancer.5821 (PMC5392208; doi:10.2196/cancer.5821)
Supplement: Multimedia Appendix 1 [file cancer_v3i1e3_app1.pdf]

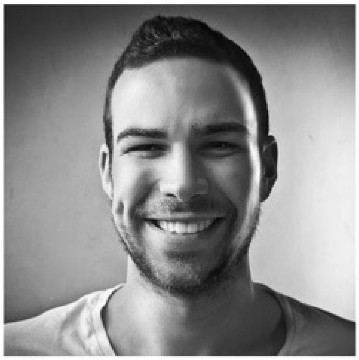
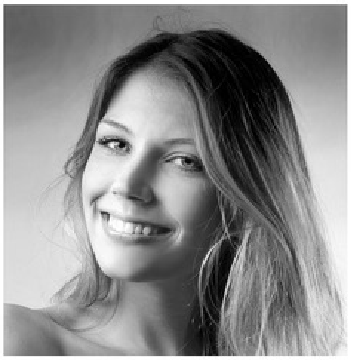
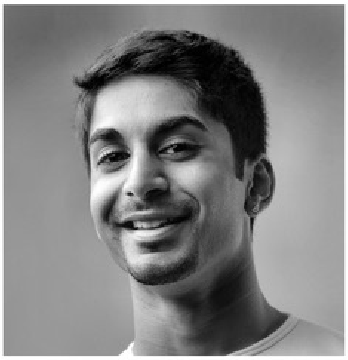
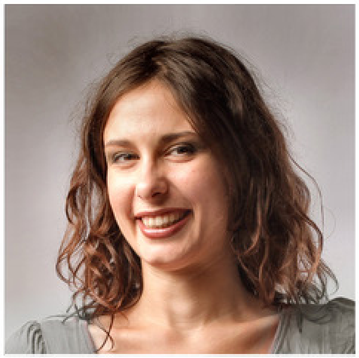

ACCESS AYA PEARLS

CHILDHOOD, ADOLESCENT AND YOUNG ADULT (AYA) CANCER SURVIVORS: CHRONIC LATE EFFECTS

One in 640 young adults between the ages of 20 and 39 is a survivor of childhood cancer. [1]
Nearly 70,000 young adults between the ages of 15-39 are diagnosed each year in the US. [2]

EVIDENCE ASSESSMENT

- Medical and psychological long-term consequences of cancer treatment are referred to as "late effects."
- 62.3 % of young adult survivors of childhood cancers have at least one late effect of cancer treatment and 27.5% suffer a severe or life-threatening late effect.[3]
- Childhood and young adult survivors face increased rates of death as a result of subsequent malignancies, cardiovascular and pulmonary disease.
- Realize that you can, and will, see diseases of older people in this younger population.
- Many childhood and AYA cancer survivors are not receiving appropriate risk-based follow-up care.

WHAT SHOULD I DO WITH THIS INFORMATION?

1. Take steps to Identify AYA and childhood cancer survivors among your patient panel.
2. Take a risk-based approach to evaluation of individual survivors patient
3. Have a high index of suspicion, and be alert for: secondary malignancies (such as breast cancer, leukemias, and sarcomas), cardiac dysfunction, and endocrinopathies
4. Recognize these patients may have poorer psychosocial health, fertility and health maintenance.
5. Recognize potential barriers to care including survivors' lack of knowledge about late effects and unawareness of risks, lack of insurance and financial resources.
6. Refer survivors and family members to resources including Seton Survivor Center, LIVESTRONG, Cancer Care and other regional and national resources.
7. Work with your survivor patients to develop personalized survivorship treatment and care plans that include recommended screening and medical care (both cancer specific and generalized health maintenance) as well as psychosocial resources.

WANT MORE INFORMATION?

Children's Oncology Group, ed. Long Term Follow-Up Guidelines for Survivors of Childhood, Adolescent and Young Adult Cancers.

Arcadia CA: Children's Oncology Group 2006.

www.survivorshipguidelines.org

Seton Cancer Survivor Center Nurse Navigator

Tel: 512.324.3343

www.seton.net/survivorship

AYA Healthy Survivorship iPhone app and Cancer Survivorship Plans

www.healthysurvivorship.org

PEARLS = Prompt Evidence Assessment and Review of the Literature Service

REFERENCES

1. Hewitt M, Weiner SL, Simone JV. (2003) Childhood Cancer Survivorship: improving care and quality of life. Washington DC: The National Academies Press

2. American Cancer Society (2012) Cancer Treatment and Survivorship Facts & Figures 2012-2013

3. Oeffinger KC, Mertens AC, Sklar CA, Kawashima T, Hudson MM, Meadows AT et al.(2006) Chronic health conditions in adult survivors of childhood cancer. N Engl J Med. 355(15):1:572-1582.

PCP Responsibility

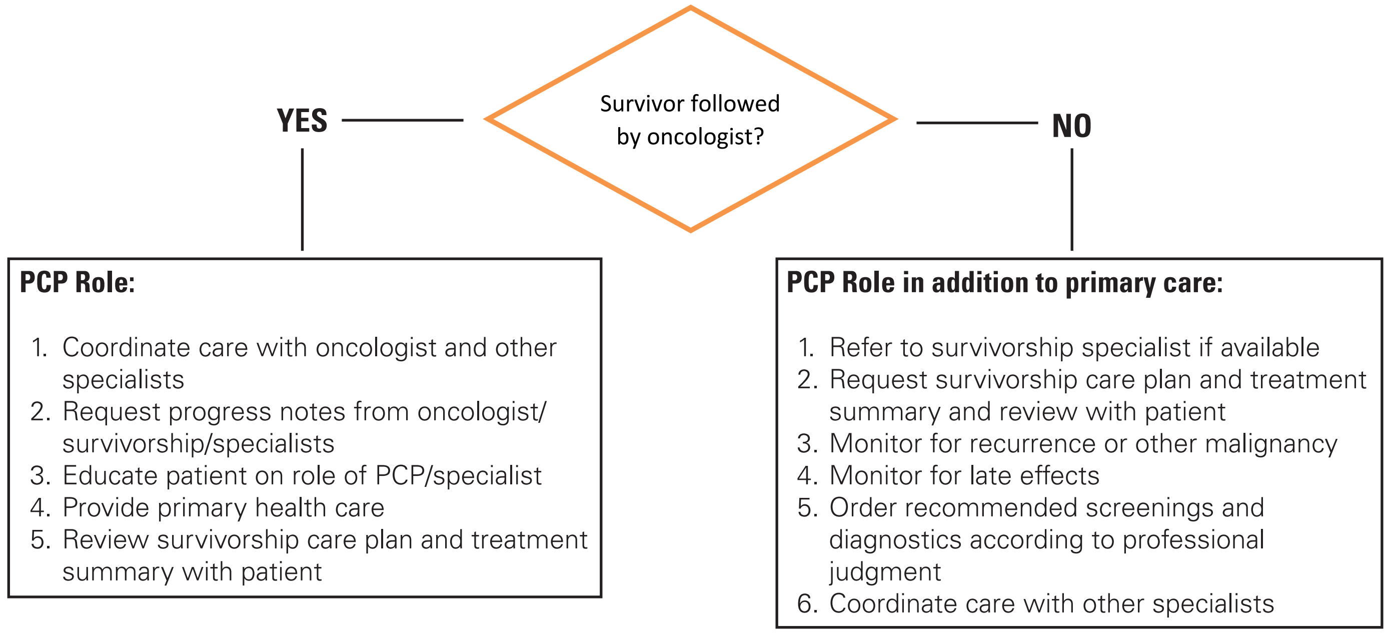

REFERRALS

The Seton Cancer Survivor Center sees patients ages 18-39 that have completed cancer treatment. Patients may call 512-324-9652 and choose to speak with the nurse navigator, or you may call the nurse navigator directly (512) 324-3343.

Referrals may be emailed to aya-survivorship@seton.org or faxed to (512) 406-6515.

More information is available at http://seton.net/survivorship.

ACCESS AYA PEARLS SPONSORED BY

Seton Healthcare Family

ACCESS AYA

CPRIT
CANCER PREVENTION & RESEARCH INSTITUTE OF TEXAS

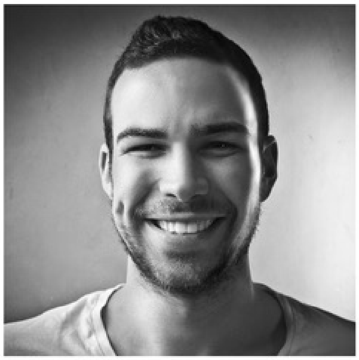
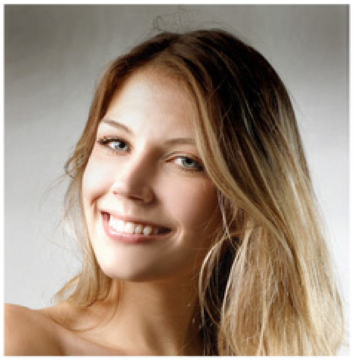
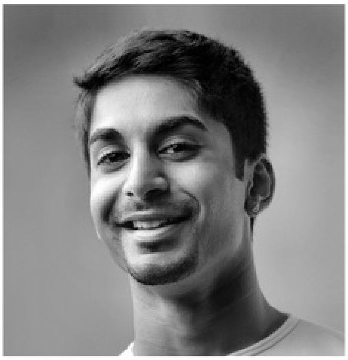
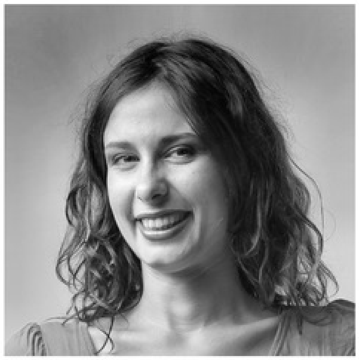

ACCESS AYA PEARLS

PSYCHOLOGICAL, SOCIAL, AND BEHAVIORAL ISSUES OF ADOLESCENT AND YOUNG ADULT (AYA) CANCER SURVIVORS

Compared to people who have never had cancer, AYA cancer survivors are more likely to smoke, less likely to get enough physical activity, and less likely to be able to afford to visit a doctor.[1]

AYAs experience a wide range of physical, psychological, social and spiritual concerns, similar to all cancer survivors. However, they often have unique needs due to their cancer occurring during a crucial stage of their personal and social development.[2]

EVIDENCE ASSESSMENT

- Some long-term AYA cancer survivors report poorer health outcomes including higher rates of obesity, anxiety and depression. [3]
- Some AYA survivors experience cognitive impairment, which can impact on employment and educational attainment. [4]
- Concerns related to body image, reduced fertility and sexual dysfunction are also prevalent among AYA cancer survivors.[5]
- Many childhood cancer survivors are not receiving appropriate risk-based follow-up care.

WHAT SHOULD I DO WITH THIS INFORMATION?

1. Take steps to Identify AYA and childhood cancer survivors among your patient panel.
2. Recognize potential barriers to care including survivors' lack of knowledge about late effects, low health literacy, lack of insurance and financial resources.
3. Refer survivors and family members to resources including Seton Survivor Center, LIVESTRONG, Cancer Care and other regional and national resources.
4. Encourage your survivor patients to develop a personalized survivorship care plan that includes recommended screening and medical care (both cancer specific and generalized health maintenance) as well as psychosocial resource.

WANT MORE INFORMATION?

Children's Oncology Group, ed. Long Term Follow-Up Guidelines for Survivors of Childhood, Adolescent and Young Adult Cancers.

Arcadia CA: Children's Oncology Group 2006.

www.survivorshipguidelines.org

Seton Cancer Survivor Center Nurse Navigator

Tel: 512.324.3343

www.seton.net/survivorship

AYA Healthy Survivorship iPhone app and Cancer Survivorship Plans

www.healthysurvivorship.org

PEARLS = Prompt Evidence Assessment and Review of the Literature Service

REFERENCES

1. Tai E, Buchanan N, Townsend J, Fairley T, Moore A, Richardson LC. (2012) Health status of adolescent and young adult cancer survivors. Cancer, American Cancer Society.

2. Clinton-McHarg et al. (2010) Measuring the psychosocial health of adolescent and young adult survivors AYA) cancer survivors: a critical review. Health and Quality of Life Outcomes. 8:25

3. Hedstrom M, Kreuger A, Ljungman G, Nygren P, von Essen L: Accuracy of assessment of distress, anxiety, and depression by physicians and nurses in adolescents recently diagnosed with cancer. Pediatric Blood & Cancer 2006, 46(7): 773-779.

4. Langeveld NE, Stam H, Grootenhuis MA, Last BF. (2002) Quality of life in young adult survivors of childhood cancer. Supportive Care in Cancer 10(8): 579-600.

5. Pacey AA (2007) Fertility issues in survivors from adolescent cancers. Cancer Treat Rev. 2007 Nov;33(7):646-55.

PCP Responsibility

Survivor followed by oncologist?

YES

PCP Role:

1. Coordinate care with oncologist and other specialists
2. Request progress notes from oncologist/ survivorship/specialists
3. Educate patient on role of PCP/specialist
4. Provide primary health care
5. Review survivorship care plan and treatment summary with patient

NO

PCP Role in addition to primary care:

1. Refer to survivorship specialist if available
2. Request survivorship care plan and treatment summary and review with patient
3. Monitor for recurrence or other malignancy
4. Monitor for late effects
5. Order recommended screenings and diagnostics according to professional judgment
6. Coordinate care with other specialists

REFERRALS

The Seton Cancer Survivor Center sees patients ages 18-39 that have completed cancer treatment. Patients may call 512-324-9652 and choose to speak with the nurse navigator, or you may call the nurse navigator directly (512) 324-3343.

Referrals may be emailed to aya-survivorship@seton.org or faxed to (512) 406-6515.

More information is available at http://seton.net/survivorship.

ACCESS AYA PEARLS SPONSORED BY

Seton Healthcare Family

ACCESS AYA

CPRIT
CANCER PREVENTION & RESEARCH INSTITUTE OF TEXAS

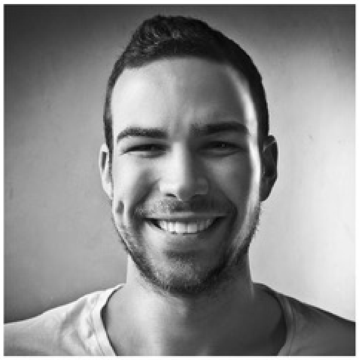
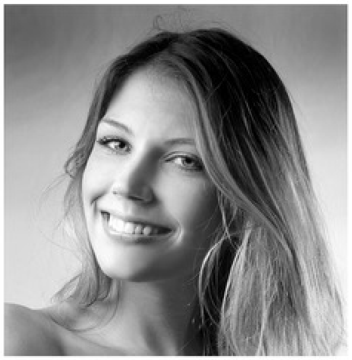
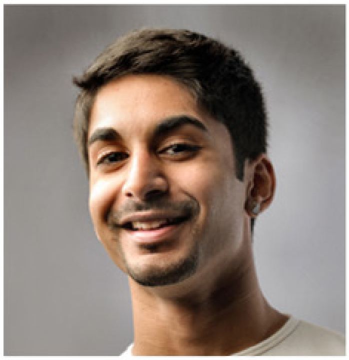
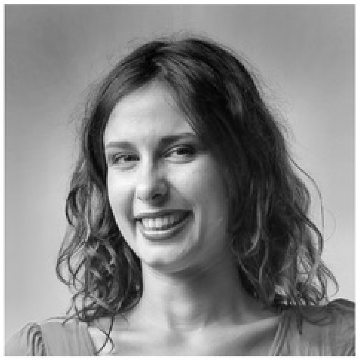

ACCESS AYA PEARLS

CHILDHOOD, ADOLESCENT AND YOUNG ADULT (AYA) CARDIOTOXIC CHRONIC LATE EFFECTS

One in 640 young adults between the ages of 20 and 39 is a survivor of childhood cancer. [1]
Nearly 70,000 young adults between the ages of 15-39 are diagnosed each year in the US. [2]

THE EVIDENCE

- Compared with those who have never had cancer, more AYA survivors had heart disease (14% vs.7%) and high blood pressure (35% vs. 29%); these conditions may be long term effects of being treated for cancer as a child or young adult. [1]
- Anthracycline-based therapy has correlated with an increased risk of cardiac disease, and expert panels recommend that AYA and childhood cancer survivors who received anthracyclines be monitored for cardiac disease. [2, 3]
- Many other chemotherapy agents, as well as new oral "targeted" agents may have cardiotoxic side effects, but these are more likely to be acute, rather than long term effects. [4, 5, 6]
- Female gender has also been associated with increased risk for cardiac disease in several studies. The reason female gender has been correlated with this risk is unknown. [7]
- Mediastinal radiation, radiation to the lungs, left abdomen and treatment for Wilms Tumor and Ewing's Sarcoma may raise concerns for cardiac issues. [8]

WHAT SHOULD I DO WITH THIS INFORMATION?

1. Take steps to identify AYA and childhood cancer survivors among your patient panel.
2. A detailed cardiac assessment should be performed for survivors of childhood cancer and AYAs who are pregnant or planning a pregnancy or who wish to take part in competitive sports.
3. Recognize potential barriers to care including survivors' lack of knowledge about late effects and unawareness of risks, low health literacy, lack of insurance and financial resources.
4. Refer survivors and family members to resources including Seton Survivor Center, Seton Heart Institute's Cardio-Oncology Program, LiveSTRONG, Cancer Care and other regional/national resources.
5. Encourage your survivor patients to develop a personalized survivorship care plan that includes recommended screenings (both cancer specific and recommended health screenings as well as psychosocial resources and needs.

WANT MORE INFORMATION?

Children's Oncology Group, ed. Long Term Follow-Up Guidelines for Survivors of Childhood, Adolescent and Young Adult Cancers.

Arcadia CA: Children's Oncology Group 2006.

www.survivorshipguidelines.org

Seton Cancer Survivor Center Nurse Navigator

Tel: 512.324.3343

www.seton.net/survivorship

AYA Healthy Survivorship iPhone app and Cancer Survivorship Plans

www.healthysurvivorship.org

REFERENCES

1. Tai E, Buchanan N, Townsend J, Fairley T, Moore A, Richardson LC. (2012) Health status of adolescent and young adult cancer survivors. Cancer.

2. Mulroney DA, Yeazel MW, Kawashima T, et al. (2009) Cardiac outcomes in a cohort of adult survivors of childhood and adolescent cancer: retrospective analysis of the Childhood Cancer Survivor Study cohort. BMJ 2009; 339: b 4606.

3. J. Leandro, J. Dyck, D. Poppe et al.1994) "Cardiac dysfunction late after cardiotoxic therapy for childhood cancer," American Journal of Cardiology 74:11. 1152–1156.

4. Hinkle AS, Proukou CB, Deshpande SS. Cardiovascular Complications: Cardiotoxicity Caused by Chemotherapy. In: Wallace H Green DM, Eds. Late effects of Childhood Cancer New York. Oxford University Press; 2004895-100.

5. Green DM, eds. (2000) Late Effects of Childhood Cancer. New York: Oxford University Press; 2004:85-100. V. B. Pai and M. C. Nahata, "Cardiotoxicity of chemotherapeutic agents. Incidence, treatment and prevention," Drug Safety .22:4. 263:302.

6. Garcia-Alvarez, X. Garcia-Albeniz, J. Esteve, M. Rovira, and X. Bosch,(2010) "Cardiotoxicity of tyrosine-kinase-targeting drugs," Cardiovascular and Hematological Agents in Medicinal Chemistry. 8:1.11:21.

7. Green, YA. Grigoriev, B. Nan et al., "Congestive heart failure after treatment for Wilms' tumor: a report from the National Wilms' Tumor Study Group," Journal of Clinical Oncology 2001. 19:7.1926:934.

8. Hull MC, Morris CG, Pepine CJ, Mendenhall NP.(2003) Valvular dysfunction and carotid, subclavian, and coronary artery disease in survivors of Hodgkin lymphoma treated with radiation therapy. JAMA 2003; 290 2831-7.

REFERRALS

The Seton Cancer Survivor Center sees patients ages 18-39 that have completed cancer treatment. Patients may call 512-324-9652 and choose to speak with the nurse navigator, or you may call the nurse navigator directly (512) 324-3343.

Referrals may be emailed to aya-survivorship@seton.org or faxed to (512) 406-6515.

More information is available at http://seton.net/survivorship.

ACCESS AYA PEARLS SPONSORED BY

Seton Healthcare Family

ACCESS AYA

CPRIT
CANCER PREVENTION & RESEARCH INSTITUTE OF TEXAS
